# Experimental investigation of rotating nodal line of MEMS-based nonlinear multi-mode resonators

**DOI:** 10.1038/s41598-022-26014-3

**Published:** 2022-12-09

**Authors:** Chun-You Liu, Sheng-Shian Li

**Affiliations:** 1 Department of Power Mechanical Engineering, National Tsing Hua University, Hsinchu City, Taiwan; 2 Institute of NanoEngineering and MicroSystems, National Tsing Hua University, Hsinchu City, Taiwan

**Keywords:** Engineering, Physics

## Abstract

Nonlinear phenomenon is presently attracting considerable attention in the field of microelectromechanical systems (MEMS). By adjusting a controllable tuning voltage, the nonlinearity of microdevices, especially on microactuators, can be precisely manipulated. To trap and separate small particles, generating a large and stable rotation force is critical in micromanipulations. Here, we report a simple and potential angular momentum cell comprising a piezoelectric MEMS-based nonlinear multi-mode resonator with integrated electrodes. A nonlinear rotating nodal line has been observed in specific frequency bands by applying a controllable low voltage of sub 5 V on a 4-port resonator made of lead zirconate titanate (PZT) thin films. The magnitude of the actuated voltage is Complementary-Metal-Oxide-Semiconductor (CMOS)-compatible and easy to integrate with the circuit. Furthermore, the real-time rotation motion of the MEMS-based nonlinear multi-mode resonator is also verified by a laser doppler vibrometer (LDV) at both chirp and single input frequencies, respectively. Therefore, this angular momentum cell shows great potential in the application of micromanipulation.

## Introduction

MEMS sensors and actuators are utilized for the domains from optical/electrical applications such as spectroscopy^1^ and frequency synthesizers^2^ to the automotive industry such as inertial sensors^3^, medical devices such as microfluidics^4^ and biomechanical transducers^5^, and many others. In order to simplify the operation of MEMS devices, most MEMS devices work in the linear region. Hence, the performance of MEMS devices can be predictable and controllable. Nevertheless, due to the requirements of the applications, some MEMS devices need to operate under a large driving force, which leads to the nonlinearity of MEMS devices that cannot be ignored. Therefore, the inherently strong nonlinearity in MEMS devices has paramount importance and the study of nonlinear behavior in MEMS devices has been actively investigated for decades. Recently, nonlinear phenomena are widely used to improve the performance of sensors and actuators by exploiting their nonlinear characteristic behavior of intrinsic nonlinearity caused by material properties and exterior nonlinearity caused by geometry. For instance, (1) in optics, harmonic generators and modulators are using nonlinear phenomena to generate frequency combs^6^, higher harmonics^7^, and parametric oscillations^8^; (2) in Physical MEMS, parametric modulation is also recognized as an efficient way to enhance the quality factor (*Q*-factor) as well as signal-to-noise ratio (SNR) of microresonators^9^; (3) in BioMEMS, small particles in the field of fluidics can be captured and separated by using the technology of micromanipulation^10,11^.

There are several significant nonlinearity phenomena at the micro scale such as Duffing effect caused by second-order nonlinear behavior^12^, the parametric effect caused by the generation of multiple coherent integer frequencies^13^, and internal resonance caused by nonlinear energy transfer from Eigenmodes^14^. In literature, to investigate the Duffing effect in resonant devices, a chirp driving signal is used to excite MEMS devices into the nonlinear region, and the trace of the Duffing curve is recorded by electrical or optical measurements; then parametric effects of MEMS resonators are usually characterized by electrical measurement such as spectrum analyzer where multiple coherent integer frequencies over a wide frequency span are recorded; finally, once the Eigenmodes have the relationship of integer frequencies, the internal resonance, i.e., nonlinear energy transfer, will take place at a specific frequency with a threshold voltage. This nonlinear phenomenon can be usually observed by electrical measurements such as a network analyzer (NA) and optical measurements such as laser doppler vibrometer (LDV). Nevertheless, most research on nonlinear behaviors in literature has been characterized by electrical measurement in the frequency domain. The time-dependent nonlinear behavior such as circularly polarized mechanical resonances is rarely explored^15,16^. In general, the time-dependent nonlinear rotation motion can be actuated based on the frictional coupling regarding nonlinear subharmonic traveling resonant modes^17^ or acoustic streaming based on the acoustic radiation torque^18^. Compared to the previous work, the rotation motion based on the nonlinear multi-mode coupling is experimentally validated in this work. Through real-time electrical and optical measurements, we investigate the rotating nodal line of a piezoelectric MEMS-based nonlinear multi-mode resonator with integrated electrodes. There is no intrinsic mechanical rotation motion of Eigenmodes in the linear region. The intrinsic nonlinear behavior in this work is dominated by dielectric and piezoelectric nonlinearities provided by soft PZT. To actuate the nonlinear behavior of this PZT-based membrane resonator, a 4-port scheme is proposed to excite the normal (1,1) mode of this membrane resonator. When the chirp or sinusoidal driving signal exceeds the threshold level, the mechanical rotation motion will take place at specific frequencies. This resonator is operated at ambient temperature with CMOS-compatible threshold voltage and the rotational motion is recorded by LDV. As a result, this experimental validation of a nonlinear MEMS resonator with integrated electrodes shows great potential in the application of micromanipulation.

## Results

### Design concept and applications

**Figure 1:**
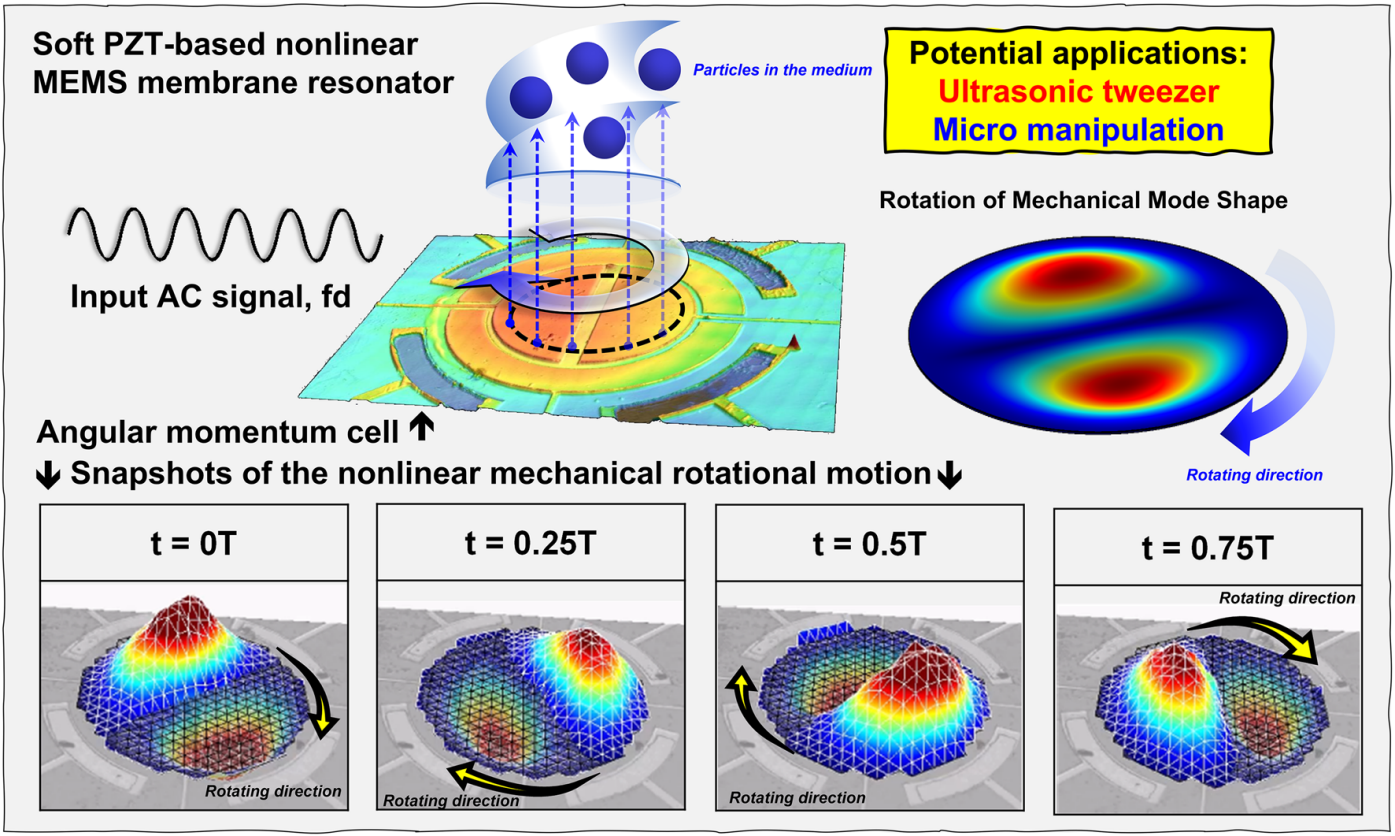
Schematic of the proposed piezoelectric nonlinear MEMS resonator-based angular momentum cell: A CMOS-compatible input electrical signal is applied to the piezoelectric device and triggers the mechanical rotation motion. To identify the real-time motion, snapshots of the clockwise rotation motion are provided.

It is well known that an elastic circular membrane possesses pairs of degenerate modes, which have the same linear natural frequencies and mode shapes^19^. Through the theoretical analysis regarding discrete nonlinear partial differential equations of motion in a multi-mode nonlinear membrane resonator, there are two subharmonic motions, subharmonic standing wave (SSW) and subharmonic traveling wave (STW), which may be triggered simultaneously caused by the nonlinear coupling between modes^20^^,^^21^. The numerical solution shows that STW bifurcates from SSW solutions and loses stability via Hopf bifurcation. For sufficiently large values of the frequency detuning, the STW, which is superposition of periodic motions with rotating nodal lines and periodic motions at the frequency of the driving, takes place. This type of motion is giving rise to quasi-periodic motions called mechanical rotation motion in this work^22^. To investigate this nonlinear phenomenon, the device utilizes a soft PZT-based membrane resonator and integrated electrodes which provide 4 multiple input and output (Multi-I/O) ports serving as a potential angular momentum cell shown in Fig. 1. The profile of the proposed resonator is captured by Keyence confocal microscope^23^. To characterize the mechanical motion of this membrane resonator, an optical measurement was performed by LDV. The mechanical motion is actuated by applying a single-tone drive signal (*f*_*d*_) (beyond the linear resonant frequency) to the inner electrode of the resonator with an amplitude high enough to drive the resonator into its strong nonlinearity. As the drive signal exceeds a threshold voltage, the rotational motion of a MEMS-based nonlinear multi-mode resonator is triggered and then such a rotational motion was observed and recorded by LDV. Therefore, as shown in Fig. 1, the real-time periodic rotation motions are divided into four time segments through snapshots which include starting time (0 T), quarter period (0.25 T), half period (0.5 T), and three-quarters period (0.75 T). A clockwise rotation motion with a period of 1/*f*_*d*_ ensues a standing wave with a gradient in an out-of-plane direction, which provides a stable rotation torque. It is worth noting that the magnitude of the actuated voltage is CMOS-compatible and easy to integrate with the circuit. As a result, this nonlinear behavior of the mechanical rotation motion shows great potential in micromanipulations and other applications.

### Nonlinear behavior characterization under frequency sweep conditions

**Figure 2:**
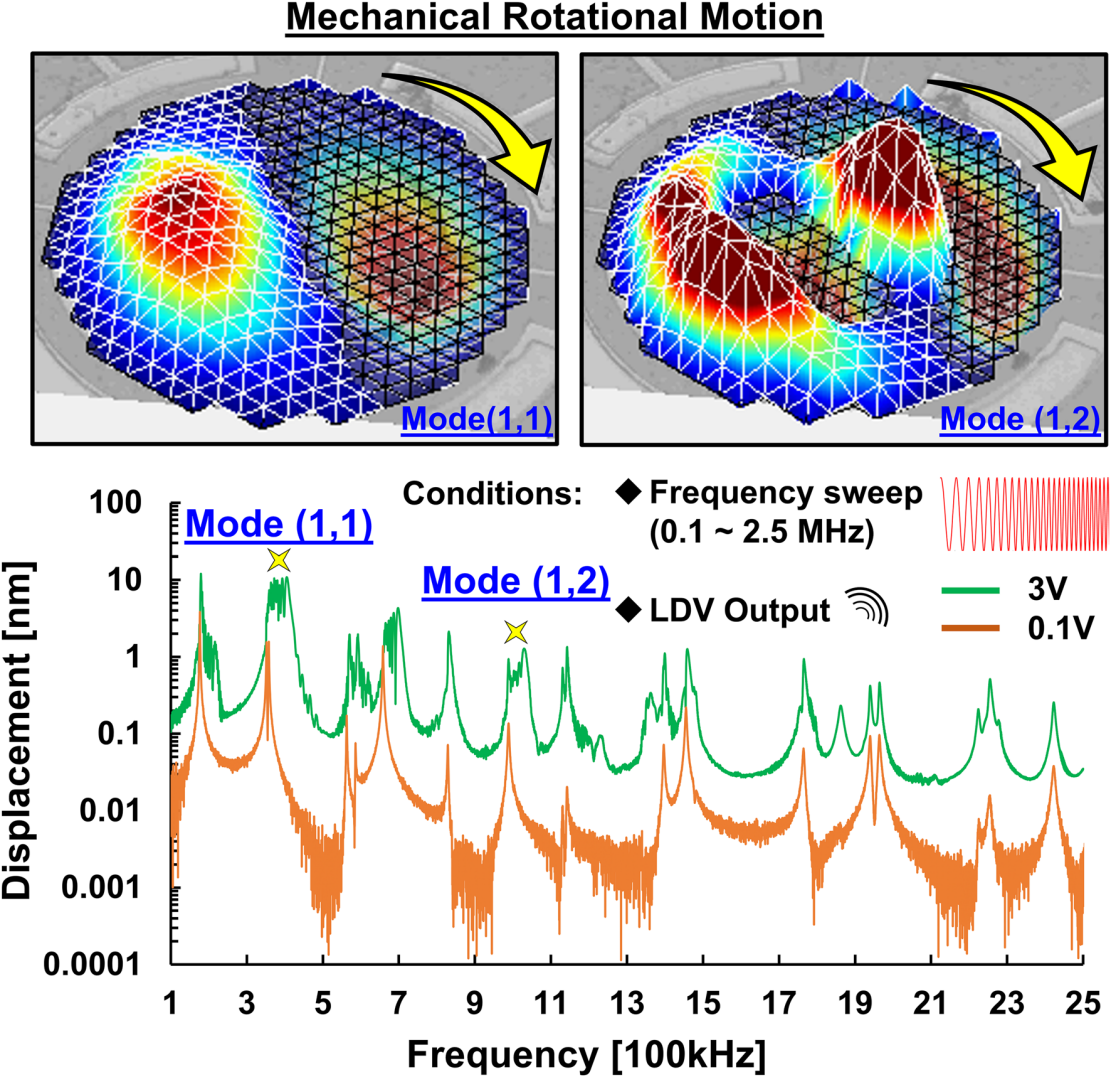
Comparison of the Eigen and rotation motions characterized by LDV measurement: Two mechanical rotation motions are observed by an actuated chirp signal (frequency range from 0.1 to 2.5 MHz) with a sufficiently high voltage of 3 V to drive the resonator into a strong nonlinear state. The measurement results of different linear and nonlinear states are presented for comparison.

In general, LDV can identify the mechanical response of MEMS devices under frequency sweep conditions. By utilizing the magnitude and phase data of the resonator from LDV, the mechanical motion of MEMS devices can be verified and presented as a 3D profile^24^. To investigate the nonlinear behavior of the soft PZT-based membrane resonator, the resonator is actuated into different linear and nonlinear states by chirp driving signals of 0.1 V and 3 V which cover frequencies from 100 to 2.5 MHz, respectively, for comparison. As presented in Supplementary Video. 1, under a frequency sweep of 0.1 V, all Eigen modes have Eigen motions over a wide frequency span without mode rotation. To briefly characterize the motions of Eigenmodes in a wide frequency span, two normal modes, the (1,1) and (1,2) modes, are a list of comparative examples. While increasing the driving voltage from 0.1 to 3 V, the clockwise mechanical rotation motions are observed in both normal (1,1) mode and normal (1,2) mode. The comparison of Eigen and rotational motions are shown in Fig. 2. As a result, the mechanical rotation motion was observed, and is only triggered when driven above a certain threshold voltage.

**Figure 3:**
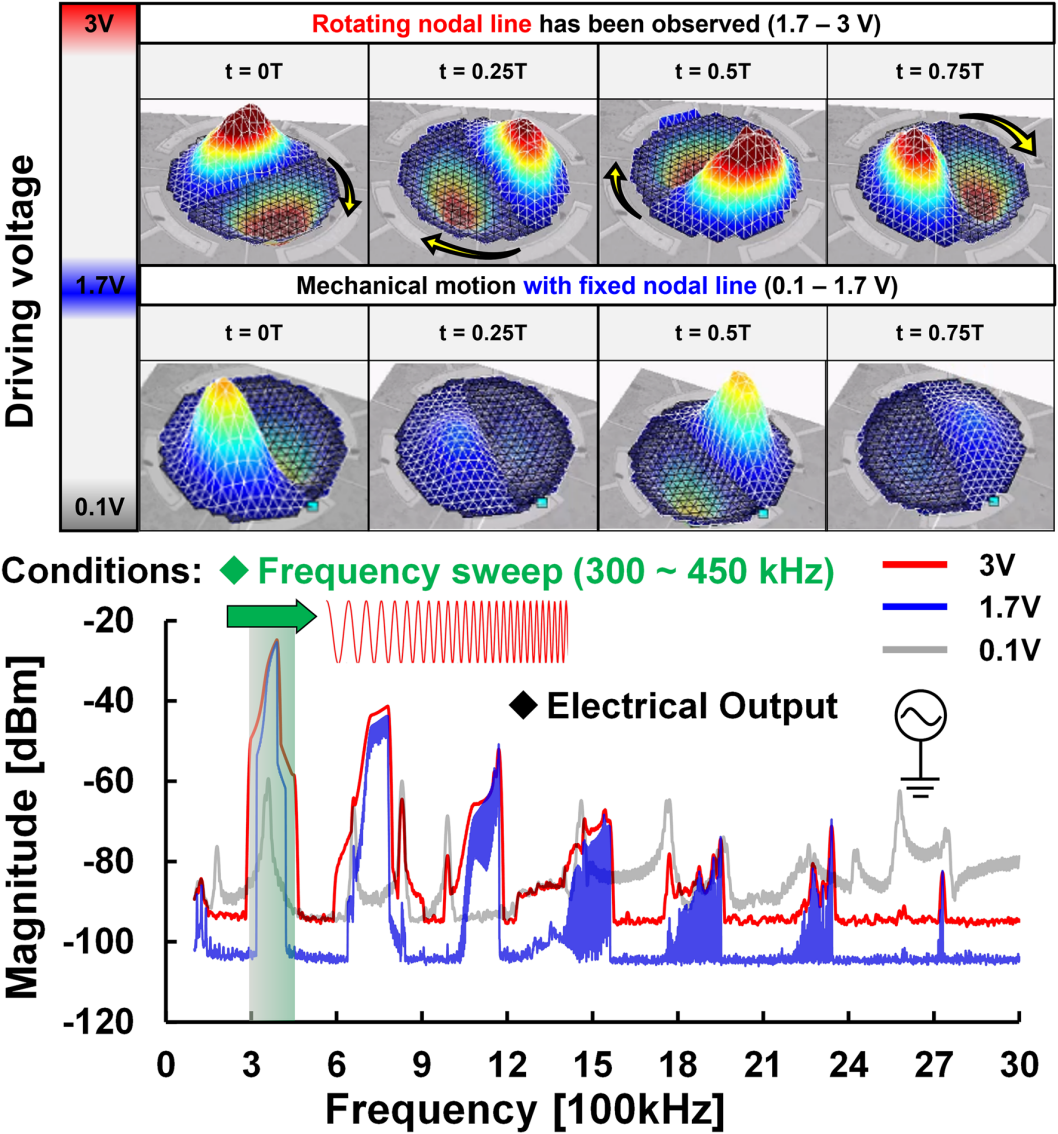
Comparison of the different driving chirp signals near desired normal (1,1) mode: The time segments of the motion of the normal (1,1) mode show two different nonlinear states representing before and after rotation. The mechanical rotation motion takes place above a threshold voltage of 1.7 V.

To confirm the threshold voltage when rotation motion occurs, a step-increasing actuated signal was given through the signal source generator. In addition, to simplify the validation of this experiment, we reduced the frequency sweep range of 2.4 MHz to a narrow band of 150 kHz near the desired frequency. Through this measurement setup, the mechanical motion of the normal (1,1) mode was captured by LDV and the electrical output was measured through the inner electrode in the spectrum analyzer (SA) using Max-hold mode simultaneously. Note that the higher-order harmonics were generated, which were induced by the nonlinear stiffness of the resonator. Once the other Eigen frequencies are close to the harmonics, the Eigen motions will be excited through the harmonics. To identify the Eigen frequencies over a wide frequency span, the measurement result is characterized by an actuated chirp signal of 0.1 V (linear operation) where frequencies from 100 to 3 MHz are used for comparison. The measurement results are shown in Fig. 3. From the result, a threshold voltage of 1.7 V that triggers the mechanical rotation was validated and the electrical frequency spectrum over a wide span was also recorded in the spectrum analyzer. Time segments of the motion of normal (1,1) mode illustrate two different nonlinear states representing before and after the rotating nodal line of the MEMS-based nonlinear multi-mode resonator taking place. Once the driving voltage pumps from 1.7 to 3 V, both the magnitude of electrical output and the rotation frequency band increase at the same time.

**Figure 4:**
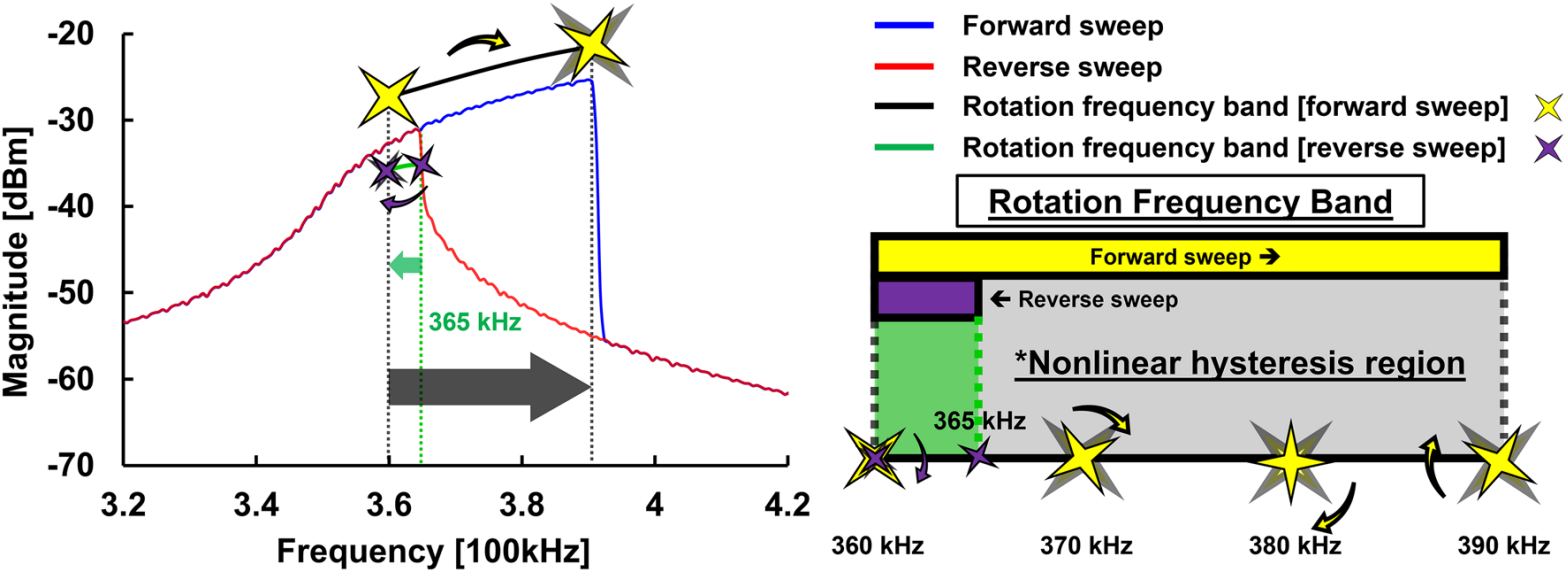
Forward and reverse frequency sweeps of the resonator: To study the rotation frequency band of the resonator, forward and reverse sweeps were performed to identify the rotation start frequency as well as the rotation stop frequency. The nonlinear rotation was observed from 360 to 390 kHz during the forward sweep. On the other hand, the nonlinear rotation was observed from 365 to 360 kHz during the reverse sweep.

Another optical measurement was performed by Digital Holographic Microscopes (DHM). DHM uses a camera to capture hologram images and videos. By reconstructing the data from DHM, a real-time 3D phase plot can be formed^25,26^. As shown in Supplementary Video. 2, an actuated chirp signal of 3 V was given through the signal source generator and the motion of the resonator was recorded in real-time by DHM. In addition, the electrical output was monitored through the inner electrode connected to the SA in Max-hold mode and used to investigate the frequency response over a wide frequency span while the quasi-periodic motion takes place. Note that the direction of the rotational motion is counterclockwise because the real-time image presented in DHM is mirror symmetric. To further identify the rotation frequency band, we examine the measurement results during the forward sweep in detail. As shown in the video, in the beginning, the mechanical motion of the resonator is in normal (1,1) mode, even though the amplitude of the driving signal (triggered voltage of 1.7 V) is high enough to drive the resonator into the nonlinear rotation as mentioned before. Once the sweep frequency reaches 360 kHz, the nodal line of the resonator starts to rotate until it reaches the non-resonant frequency of 390 kHz. Therefore, there is not only a threshold voltage but also a specific rotation frequency band to trigger the rotating nodal line of the resonator. To further study the rotation frequency band of the soft PZT-based resonator, forward and reverse frequency sweeps of the resonator were performed through the integrated inner electrode and measured using the SA shown in Fig. 4. During the forward sweep, the nonlinear mechanical rotation was observed from 360 to 390 kHz. In contrast, the nodal line of the resonator starts to rotate at 365 kHz and stops at 360 kHz during the reverse sweep. Therefore, a specific rotation frequency band from 360 to 390 kHz is identified by applying an actuated chirp signal of 3 V through the integrated inner electrode of the resonator. It is worth mentioning that the frequency band of 365 kHz to 390 kHz, usually called the nonlinear hysteresis region caused by the strong stiffness hardening effect, features the nonlinear rotation motion during the forward sweep. On the contrary, during the reverse sweep, the resonator shows normal motion, i.e., no rotation, in the nonlinear hysteresis region.

**Figure 5:**
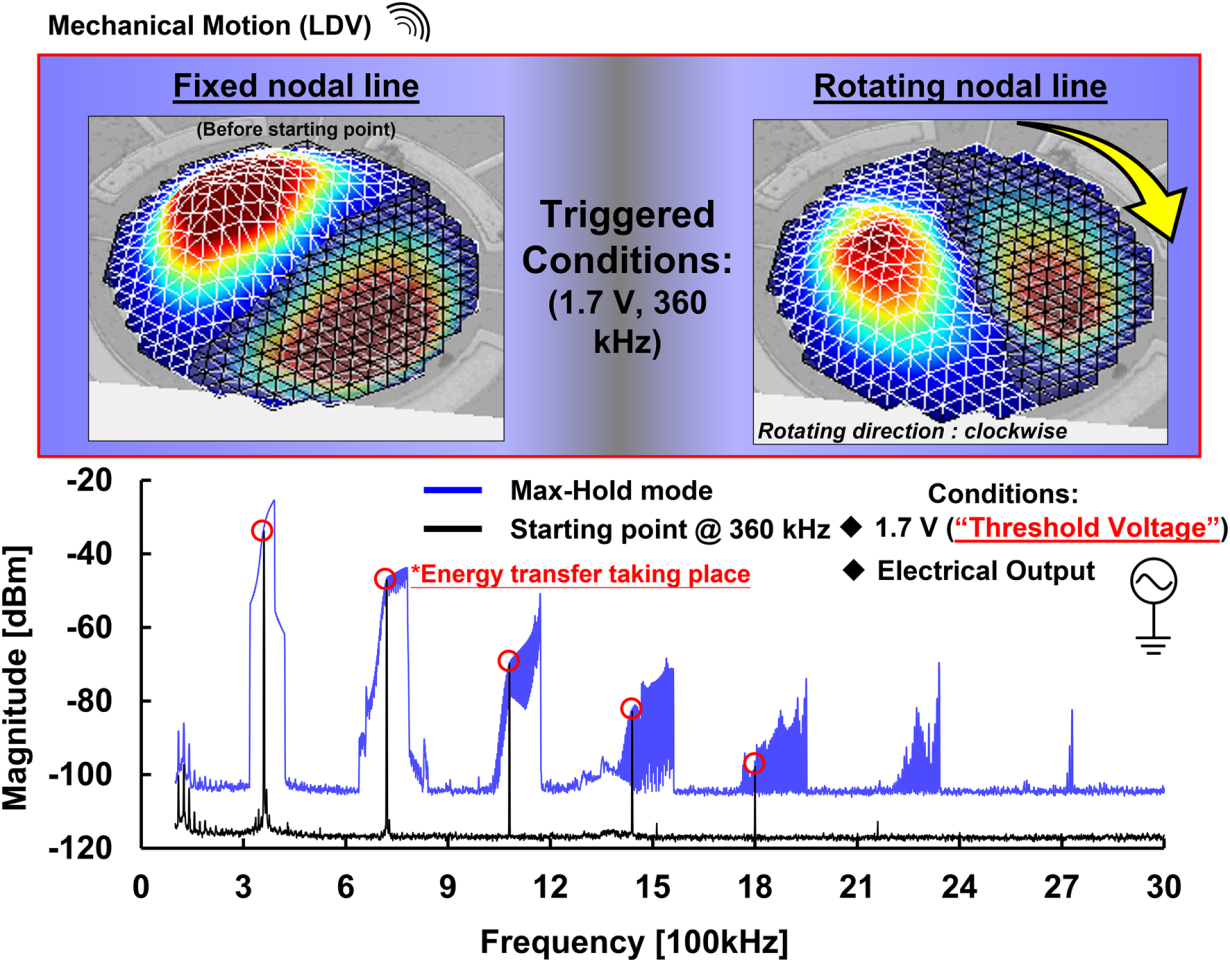
Validation of energy transfer in rotating nodal line of nonlinear MEMS resonator: To explore the energy transfer between two nonlinear energy states, electrical measurement of frequency sweep with a threshold voltage of 1.7 V was performed to be compared with a single tone driving signal at 360 kHz where the rotation takes place.

Energy transfer is one of the common behaviors in nonlinear phenomena. To explore whether energy is transferred inside the resonator through the change from the case of a fixed nodal line to the case of a rotating nodal line, a simple electrical experiment setup via a SA in Max-hold mode was performed to record the energy transfer path for validation as shown in Fig. 5. The mechanical rotation was triggered by a chirp signal with a threshold voltage of 1.7 V and measured by a SA. Furthermore, to identify the onset of the rotation, the resonator was driven with a single-tone drive signal of 360 kHz at the onset frequency of the rotation frequency band and measured in a SA for comparison. As shown in Figs. 3 and 5, the increment in the output voltage at the driving frequency (1.7 V, 360 kHz) and its integer frequencies is strong evidence of the change of the nonlinear state (from the case of fixed nodal line to the case of rotating nodal line). According to measurement results, the apparent change in the slope of magnitude at higher-order harmonics indicates that energy transfer takes place at the same time as the rotating nodal line is triggered.

To briefly explain the nonlinear behavior of the soft PZT-based MEMS resonator, we summarize the optical and electrical measurement results under various frequency sweep conditions. The nonlinear mechanical rotation has been characterized by a specific rotation frequency band via a threshold voltage of 1.7 V. Once the clockwise rotation is triggered, the energy transfer can be observed at its higher-order harmonics measured under frequency sweep conditions and recorded through a SA in Max-hold mode. Therefore, as a potential angular momentum cell, if we can simplify the operation principle of the soft PZT-based MEMS resonator from an actuated chirp signal to a single-tone driving signal, the performance of the resonator will be much easier to predict and control, which would potentially enable more easier implementations and applications using such a nonlinear rotation mechanism.

### Nonlinear behavior characterization by a single-tone driving signal

**Figure 6:**
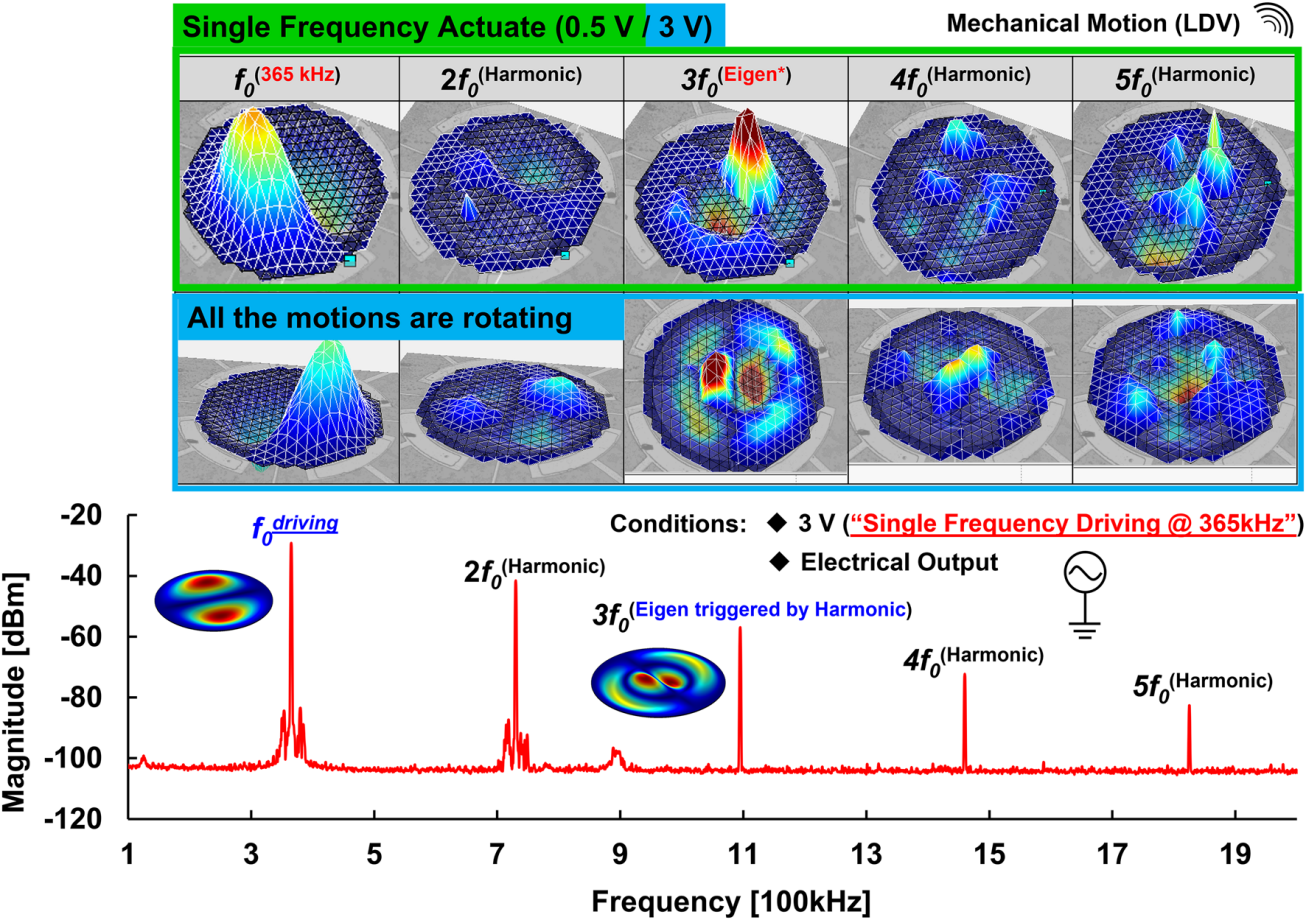
Mechanical motions with and without rotations by a single-tone driving signal of 0.5 V and 3 V, respectively: The mechanical motions were captured by LDV and driven in the rotation frequency band (at 365 kHz). The generation of higher-order harmonics and the parametric effect were also validated by the SA through the integrated inner electrode of the resonator.

As discussed in the summary of the previous section, it will be more attractive in micromanipulation applications if the nonlinear rotation mechanism can be triggered with a simple condition. To ensure that the resonator driven by a single-tone driving signal still has a threshold voltage and a specific mechanical rotation frequency span, the optical and electrical measurements provided by LDV and SA were utilized to clarify these nonlinear behaviors as shown in Fig. 6. The mechanical motions of multiple coherent integer frequencies were captured by LDV and presented in Supplementary Video. 3. A driving frequency of 365 kHz, among the rotation frequency band, with a voltage of 0.5 V, drives the resonator into the nonlinear state without rotation, which is used to excite the desired Eigenmode and its higher-order harmonics. Since the resonator was operated in the nonlinear region, common nonlinear behaviors such as the generation of higher-order harmonics can be observed in the electrical measurement through the integrated inner electrodes. As a result, the motion of *f*_*0*_ is normal (1,1) mode and most of the other coherent integer frequencies are harmonic motions induced by the higher-order stiffness of the resonator. Instead of the nonlinear behavior regarding the generation of higher-order harmonics, the motion of 3 *f*_*0*_ is normal (1,2) mode, which is excited by higher-order harmonics of driving frequency *f*_*0*_, caused by another nonlinear behavior regarding the parametric effect. In order to study the mechanical rotation by a single-tone driving signal, a higher driving voltage of 3 V is used to drive the resonator into the next nonlinear state actuating the rotating nodal line. The mechanical motions were also captured by LDV and presented in Supplementary Video. 3. Compared to the measurement result of lower voltage driving, all motions, including normal (1,1) mode, normal (1,2) mode caused by the parametric effect, and other harmonic modes, are rotating. According to the measurement results, there is still a threshold voltage required for nonlinear mechanical rotation motion in the case of a single-tone drive.

**Figure 7:**
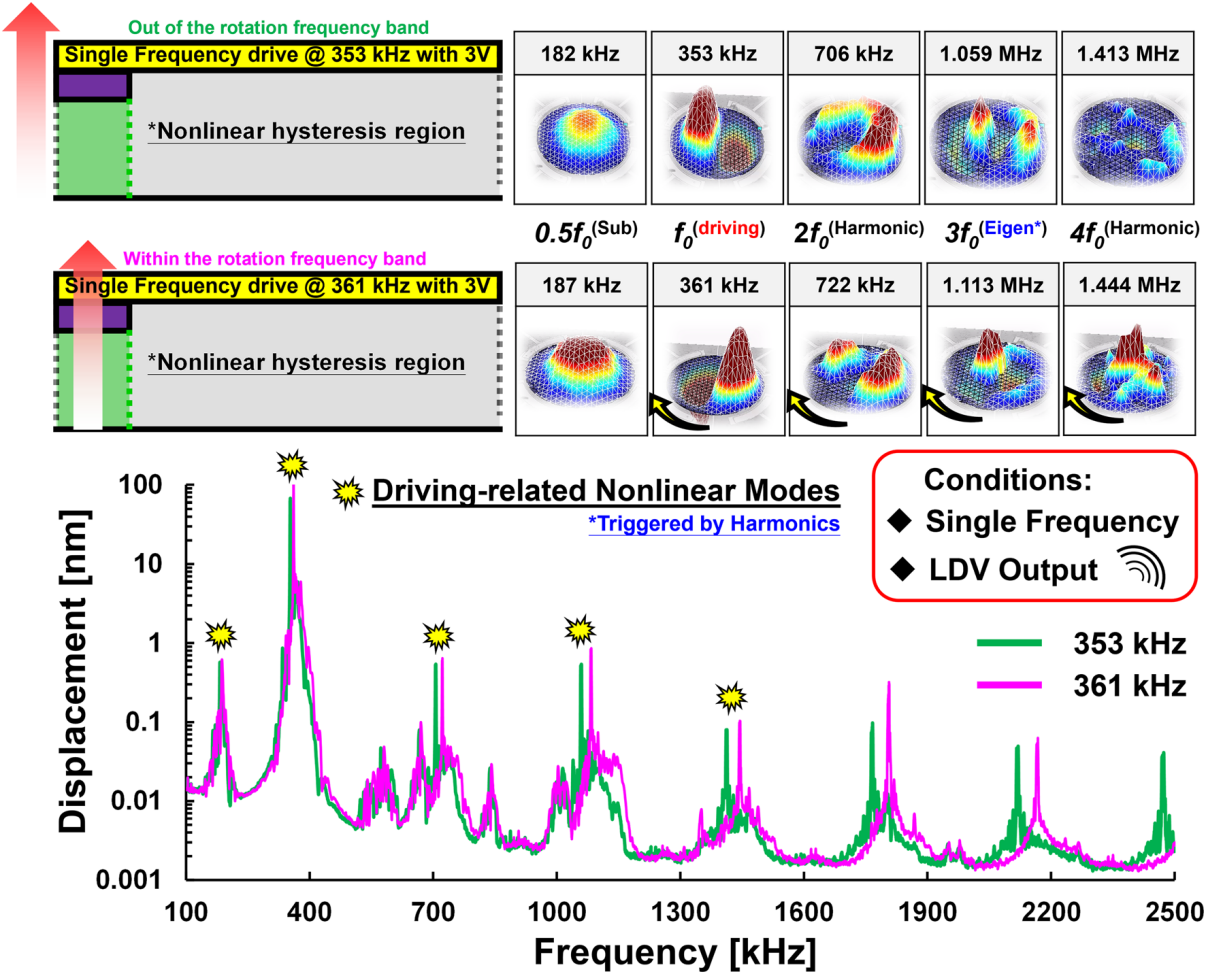
Mechanical motions driven within and out of the rotation frequency band: The mechanical motions were captured by LDV and driven at 353 kHz and 361 kHz, respectively. Note that the yellow dots identify the coherent integer frequencies and the motion at 3* f*_0_ presents the normal (1,2) mode triggered by the parametric effect.

The final validation of mechanical rotation motion under single-tone driving is on whether the rotation frequency band exists or not. To identify the different energy states, two frequencies: (a) 353 kHz (out of the rotation frequency band) and (b) 361 kHz (within the rotation frequency band), are used to drive the resonator into a strong nonlinear state for comparison. The mechanical motions were also captured by LDV and presented in Supplementary Video. 4. While driving at 361 kHz (in-band), mechanical motions of the coherent integer frequencies are all rotating. In contrast, while driving at 353 kHz (out-of-band), mechanical motions of the coherent integer frequencies only show the Eigen and harmonic motions without rotation. Figure 7 shows all the motions and displacement results by these two driving frequencies. According to the optical measurements by LDV, there still exists a rotation frequency band for the nonlinear mechanical rotation in the case of a single-tone drive.

The mechanical rotation motion provides a radiation force in the medium and shows great potential as a unit of angular momentum. The technology of micromanipulation, implemented with the angular momentum cell, has been actively investigated in microfluidics, biomedical MEMS, and powder fabrication processes. Small particles in the field of fluidics and acoustics can be captured and separated by using this technology^27,28^. These potential applications can utilize this mechanical rotation mechanism for contact-free manipulation of particles or for generating a stable rotation torque. Through the controllable and predictable trigger conditions validated in this work, this technology will be promising and attractive to be implemented in the abovementioned applications.

## Discussion

There are several nonlinear behaviors in this soft PZT-based circular membrane resonator such as the generation of higher-order harmonics, parametric effect, and the rotating nodal line of a nonlinear multi-mode MEMS resonator. Since nonlinear behaviors in MEMS devices are complicated, to investigate the nonlinear rotation phenomena, we characterize nonlinear behaviors by frequency sweep conditions and single-tone drive. According to the results of frequency sweep conditions, different energy states (including the cases of linear, nonlinear with a fixed nodal line, and nonlinear with a rotating nodal line) and a specific rotation frequency band with a threshold voltage of 1.7 V were observed by forward and reverse frequency sweep. Note that the membrane resonator presents a strong hardening stiffness and hence the motion rotates in the nonlinear hysteresis region during the forward frequency sweep because of continuous energy pumping into the devices while it does not exist during the reverse sweep. This nonlinear behavior was also validated in the case of single-tone driving. Instead of frequency sweep condition, if we use a single-tone driving signal at a frequency in the nonlinear hysteresis region or out of the rotation frequency band to actuate the resonator, the energy state will be located at the lower energy state, like during reverse frequency sweep, and mechanical motions of the resonator will be normal mode with fixed nodal line.

Since energy transfer in the nonlinear behavior, such as internal resonance and parametric effect, is a very common phenomenon, there is a nonlinear mode coupling between STW and SSW in this work. According to the results of the single-tone drive, an energy transfer path was found at all higher-order harmonics, which are driving-related nonlinear behaviors. In addition, all the mechanical motions rotate at coherent integer frequencies, accompanied by a significant slope change of amplitude at a threshold voltage of 1.7 V within a specific rotation frequency band, which implies a change of nonlinear energy states. Therefore, we can experimentally validate the nonlinear mode coupling between STW and SSW as the source of the rotating nodal line which has been numerically analyzed by Nayfeh and Vakakis groups^21^.

In this work, we experimentally present details of a specific nonlinear behavior, mechanical rotation motion, in the soft PZT-based MEMS resonator. To study this nonlinear phenomenon, real-time electrical and optical measurement setups were performed to capture and record both the mechanical motions and electrical outputs simultaneously. The change of the energy states where nonlinear rotation occurred has been completely recorded by DHM under frequency sweep conditions. In addition, the mechanical motions over a wide frequency span were captured by LDV to identify the threshold voltage and rotation frequency band for the rotating nodal line. These trigger conditions were verified by applying a chirp driving signal as well as a single-tone driving signal through the integrated inner electrodes. The proposed piezoelectric nonlinear MEMS resonator can serve as a potential angular momentum cell via a single-tone driving signal with a CMOS-compatible voltage, which leads to a variety of applications.

## Methods

### Fabrication of soft PZT-based MEMS resonators

**Figure 8:**
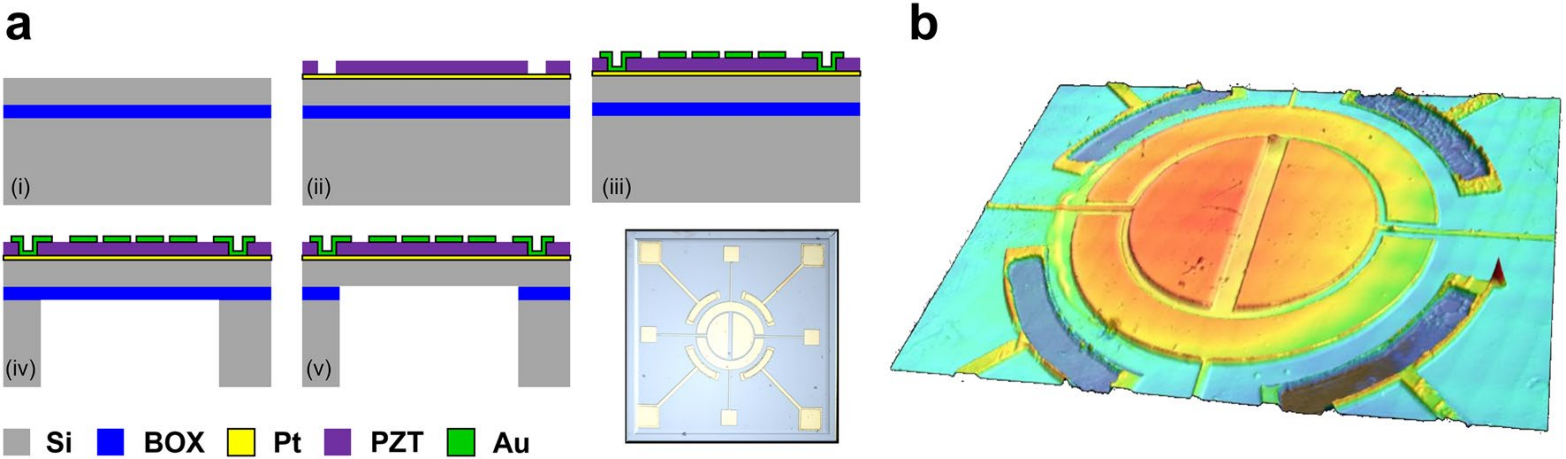
Fabrication process. (**a**) Process flow chart. (i) A 4-inch bare SOI wafer with the stacking layers of active-Si of 2 µm, buried oxide of 0.5 µm, and handle-Si of 400 µm. (ii) First mask: open via to access the bottom electrode (Pt). (iii) Second mask: top and bottom electrodes patterning by a lift-off process. (iv) Third mask: pattern backside cavity. (v) Remove the buried oxide to release the resonator. After finishing the process, the optical image was captured by an optical microscope. (**b**) The 3D profile of the resonator was measured by Keyence laser confocal.

The membrane resonators were fabricated through a three-mask microfabrication process provided by Coretronic Co. Ltd as shown in Fig. 8a. The process starts with a 4-inch silicon-on-insulator (SOI) wafer (Active-Si: 2 µm/ Buried Oxide (BOX): 0.5 µm/ Handle-Si: 400 µm). Firstly, a 150 nm platinum was deposited and serves as the bottom electrodes for the resonators. Then the soft PZT produced by the standard sol-gel process was deposited and patterned to access the bottom platinum. A 300 nm gold, which serves as the top and bottom electrodes, was fabricated by the lift-off process. Next, a backside Deep Reactive Ion Etching (DRIE) process was carried out to etch the 400 µm thick Handle-Si to form a backside cavity and the buried oxide became an etching stop layer for this dry etching process. Finally, the remaining BOX was removed by the dry etching process, and then the devices were successfully released. The final structure after the process was captured by an optical microscope shown in the inset of Fig. 8a while the laser confocal image presents the 3D profile of the resonator as shown in Fig. 8b.

### Measurement setup

**Figure 9:**
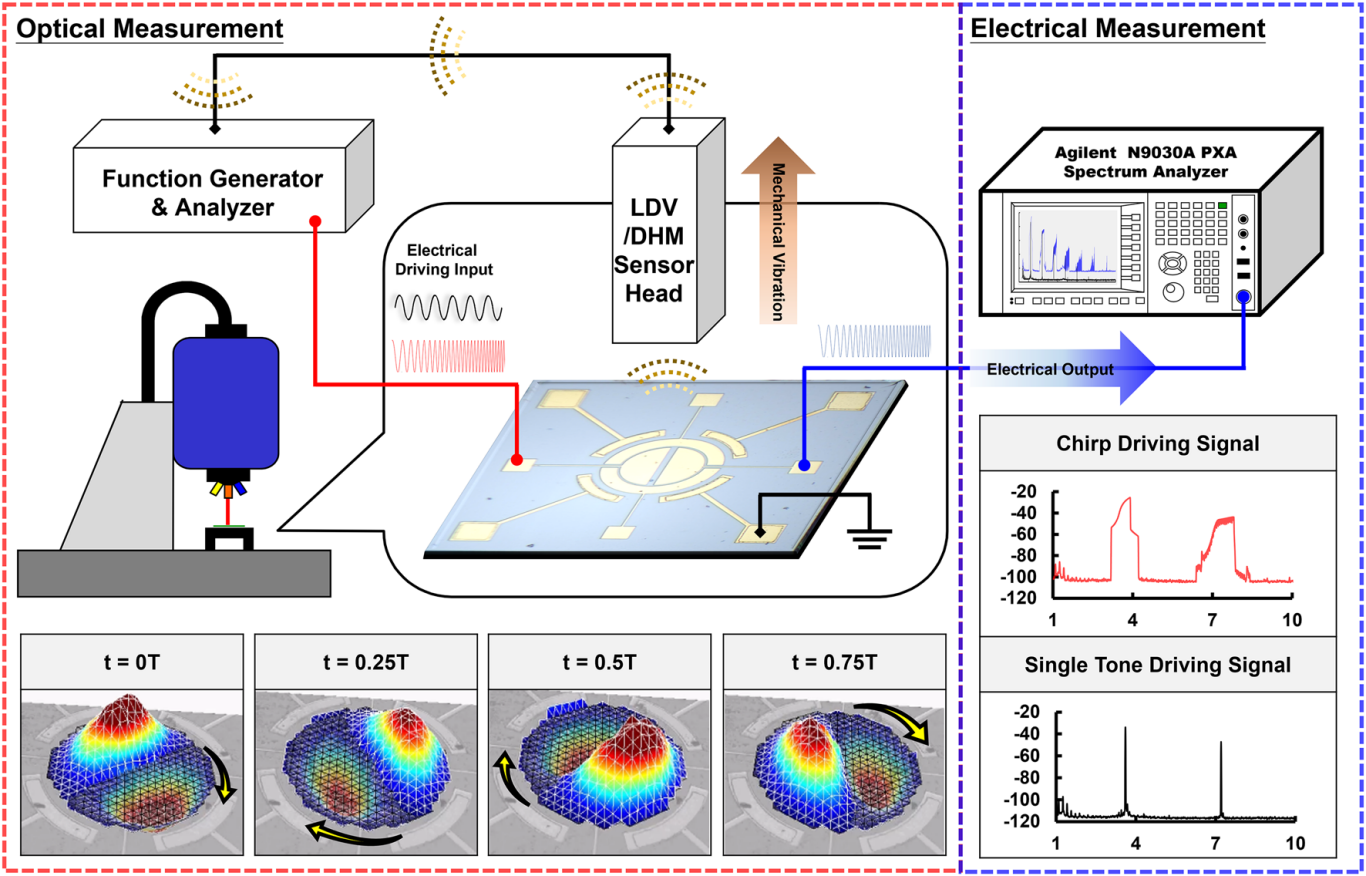
Measurement setup. The function generator provides an electrical driving input (chirp driving signal or single-tone driving signal) to actuate the resonator. The real-time mechanical vibration (Ex: the snapshots of mechanical rotation motion) can be recorded through the optical measurement performed by LDV or DHM. In addition, through the integrated inner electrodes, the electrical output can be measured by a spectrum analyzer in Max-hold mode simultaneously.

A complete measurement setup is shown in Fig. 9. Electrical outputs and mechanical vibrations can be measured at the same time. In optical measurements, electrical driving signals are provided by LDV and DHM via a function generator. Real-time vibration of the resonator can be recorded in 3D maps representing the mechanical motions. Through the snapshots of the motions, the direction of the rotation can be validated. Moreover, through the integrated inner electrodes of the resonator, electrical outputs can be measured using the spectrum analyzer. By using the Max-hold function of the spectrum analyzer, paths of the amplitude change can be recorded under frequency sweep conditions. We also can observe the harmonics over a wide frequency range through electrical measurement by the spectrum analyzer.

## Supplementary Information

Supplementary Information 1.

Supplementary Video 1.

Supplementary Video 2.

Supplementary Video 3.

Supplementary Video 4.

## Data Availability

The datasets used and/or analyzed during the current study are available from the corresponding author upon reasonable request.
