# Supplementary material for: Experimental investigation of rotating nodal line of MEMS-based nonlinear multi-mode resonators
Source: Sci Rep. 2022 Dec 9;12:21339. doi: 10.1038/s41598-022-26014-3 (PMC9734146; doi:10.1038/s41598-022-26014-3)
Supplement: Supplementary file 1 — Supplementary Information 1. [file 41598_2022_26014_MOESM1_ESM.docx]

# Supplementary materials

**Supplementary Video.1.** Comparison of the mechanical motions measured and captured by LDV under frequency sweep: The Eigen motion in the linear state and the mechanical rotation motion in the nonlinear state were actuated by a chirp signal of 0.1 V and 3V, respectively.

**Supplementary Video.2.** Real-time mechanical motion captured by DHM: The electrical output signal measured by SA was also performed at the same time. The video recorded the frequency while the mechanical rotation takes place. The rotation frequency band was also identified under the forward frequency sweep.

**Supplementary Video.3.** Comparison of the mechanical motion of the multiple coherent integer frequencies actuated by a single-tone driving signal: The mechanical rotation motions including Eigen and harmonic motions were actuated by a higher driving voltage of 3 V and captured by LDV.

**Supplementary Video.4.** Validation of rotation frequency band in rotating nodal line of the nonlinear MEMS resonator: The mechanical rotation motions including Eigen and harmonic motions were actuated by a frequency of 361 kHz (within the rotation frequency band) with the driving voltage of 3V and captured by LDV.
